# Characterisation of insomnia as an environmental risk factor for asthma via Mendelian randomization and gene environment interaction

**DOI:** 10.1038/s41598-021-01291-6

**Published:** 2021-11-08

**Authors:** Dong Jun Kim, Tae-Woong Ha, Hae Un Jung, Eun Ju Baek, Won Jun Lee, Han Kyul Kim, Ji-One Kang, Sungho Won, Ji Eun Lim, Bermseok Oh

**Affiliations:** 1 Department of Biomedical Science, Graduate School, Kyung Hee University, Seoul, South Korea; 2 Department of Biochemistry and Molecular Biology, School of Medicine, Kyung Hee University, Seoul, South Korea; 3 Department of Public Health Science, Seoul National University, Seoul, South Korea

**Keywords:** Computational biology and bioinformatics, Genetics

## Abstract

Asthma is a complex disease that is reportedly associated with insomnia. However, the causal directionality of this association is still unclear. We used asthma and insomnia-associated single nucleotide polymorphisms (SNPs) and genome-wide association study (GWAS) summary statistics to test the causal directionality between insomnia and asthma via Mendelian randomization (MR) analysis. We also performed a cross-trait meta-analysis using UK Biobank GWAS summary statistics and a gene–environment interaction study using data from UK Biobank. The interaction of genetic risk score for asthma (GRS_asthma_) with insomnia on asthma was tested by logistic regression. Insomnia was a risk factor for the incidence of asthma, as revealed by three different methods of MR analysis. However, asthma did not act as a risk factor for insomnia. The cross-trait meta-analysis identified 28 genetic loci shared between asthma and insomnia. In the gene–environment interaction study, GRS_asthma_ interacted with insomnia to significantly affect the risk of asthma. The results of this study highlight the importance of insomnia as a risk factor of asthma, and warrant further analysis of the mechanism through which insomnia affects the risk of asthma.

## Introduction

Asthma is the most common chronic respiratory disease in the world^1^ affecting people of all age groups. It is characterised by wheezing, shortness of breath, chest tightness, and coughing^2^. The prevalence of asthma has increased dramatically over the last few decades because of environmental changes^3^. Certain environmental factors, such as smoking, obesity, and air pollution, reportedly affect the incidence of asthma^4–6^.

Insomnia is a highly prevalent condition^7^ that is characterised by difficulties in initiating or maintaining sleep, or having poor sleep quality^8^. Consequently, it affects the quality of life, mood, and emotions of an individual^9^. Interestingly, an association between insomnia and asthma has been frequently reported^10–16^. Some studies suggest that asthma is associated with poor sleep quality, and is an important risk factor for developing the symptoms of insomnia^10–12^. On the contrary, there are also reports indicating that insomnia is related to upper respiratory diseases, such as asthma, and is associated with its increased risk and accompanying adverse outcomes^13–16^. As such, both causal directions seem to be plausible while exploring the association between asthma and insomnia; however, no clear causal directionality has been established between the two, and thus it warrants further investigation to elucidate their possible relationship.

Randomised controlled trials are the gold standard for inferring causal associations^17^. However, randomised controlled trials require a long time and high economic cost. As a result, Mendelian randomization (MR), a method of genetic epidemiology that uses randomly inherited single nucleotide polymorphisms (SNPs) associated with a risk factor (e.g. insomnia) as a proxy for environmental exposure, has been proposed as an alternative to assess causal inferences of an ‘exposure’ on an ‘outcome’ (e.g. asthma)^18^. The increasing availability of summary-level data from genome-wide association studies (GWAS) available in the public domain has provided a great opportunity to utilise MR for testing causality by integrating such data from different studies^19,20^.

The identification of shared genes between different traits may improve the understanding of the shared genetic mechanisms. To identify the shared genes between different traits, cross-trait meta-analysis have been employed. Since it can be performed with GWAS summary statistics, there are several advantages. Individual level genotype data is not required, and meta-analysis of different traits can improve the power of detecting cross-trait genetic effects that may not reach genome-wide significance level for single trait^21^.

Complex diseases often result from the interaction between genetic and environmental factors^22,23^. Gene-environment interactions suggest that individuals respond differently to environmental stimuli depending on their genotype profiles, or that genetic effects vary among individuals depending on their lifestyles^24^. Thus, identifying gene-environment interactions can potentially improve the risk assessment of diseases^22^, and provide clues for the underlying mechanisms of disease development.

Thus, in this study, we aim to elucidate the causality between asthma and insomnia using MR analysis, identify the shared genes between asthma and insomnia by cross-trait meta-analysis, and analyse the interaction of insomnia with genetic risk score of asthma (GRS_asthma_). Consequently, we used GWAS summary statistics for the MR analysis and the cross-trait meta-analysis, and calculated GRS_asthma_ using asthma-associated SNPs for the gene-environment interaction study.

## Methods

### Study population

The UK Biobank is a population‐based cohort of > 500,000 men and women aged 40–69 years who were recruited during 2006–2010^25^. For sample quality control (QC), we applied the filters present in the documentation by Neale lab (https://github.com/Nealelab/UK_Biobank_GWAS): (a) principal component analysis calculation filter to select un-related samples, (b) sex chromosome filter to remove aneuploidy, (c) principal component (PC) filter for European sample selection to determine British ancestry, and (d) filters to select self-reported ‘white-British’, ‘Irish’, and ‘white’ ancestries. To participate in UK Biobank, all participants informed consent in place of signed consent^26^. UK Biobank has been given ethical approval to collect participant data by the North West Multicentre Research Ethics Committee, the National Information Governance Board for Health & Social Care, and the Community Health Index Advisory Group. All methods were carried out in accordance with the relevant guidelines and regulations.

### Genotype data

Baseline imputed genotype data of 7,402,791 SNPs were available for 487,409 participants. We performed quality control analysis using PLINK^27^, based on the following exclusion criteria: SNPs with missing genotype call rates > 0.01, minor allele frequency (MAF) < 0.05, and Hardy–Weinberg equilibrium *P* < 1 × 10^–6^. Consequently, 5,661,690 SNPs were retained for further analysis.

### Inclusion and exclusion criteria for asthma case and control groups

Participants who constituted the asthma case group were identified by their answer to the question, ‘Has a doctor ever told you that you have had any of the following conditions?’. Those who replied ‘asthma’ were included, and those who answered ‘emphysema’ or ’chronic bronchitis’ were excluded. Further, participants diagnosed with chronic obstructive pulmonary disease (COPD) were also excluded. Asthma control group was defined as those who did not answer ‘asthma’, ‘rhinitis’, ‘eczema’, ‘allergy’, ‘emphysema’, and ’chronic bronchitis’ to the abovementioned question. In addition, those who had diagnostic records of hay fever, allergic rhinitis, emphysema, chronic bronchitis, and COPD, and who had J40-47 records in the ICD 10 codes, were excluded (Supporting Information).

### Classification of participants on the basis of their insomnia symptoms

To classify participants based on their insomnia symptoms, the following question was asked: ‘Do you have trouble falling asleep at night or do you wake up in the middle of the night?’. The participants were able to choose one of the following four answers: ‘never/rarely’, ‘sometimes’, ‘usually’, or ‘prefer not to answer’. This data was used to classify the participants’ insomnia states, and those who answered ‘prefer not to answer’ were excluded. Consequently, we transformed these categorical variables into quantitative variables as follows: 0 = ‘never/rarely’, 1 = ‘sometimes’, and 2 = ‘usually’ for further analysis.

### Mendelian randomization (MR) analysis

To assess the causal association between insomnia (exposure) and asthma (outcome), we extracted 202 insomnia-associated SNPs at genome-wide significance (*P* < 5 × 10^–8^) from 1,331,010 individuals in the UK Biobank and 23andMe consortium (Fig. S1)^28^. We used the asthma GWAS summary statistics provided by TAGC (summary data code: ebi-a-GCST006862)^29^ for the primary analysis, and then used the summary statistics provided by Neale lab (www.nealelab.is/uk-biobank; summary data code: ukb-a-446) for the replication analysis (Fig. S1). In contrast, to assess the causal association between asthma (exposure) and insomnia (outcome), we selected asthma-associated SNPs at genome-wide significance (*P* < 5 × 10^–8^) as reported in 9 GWASs^30–38^. A total of 168 independent SNPs were selected after linkage disequilibrium-based clumping (r^2^ < 0.1, kb > 250) using PLINK (Table S1). For insomnia, we used the GWAS summary statistics provided by the Neale lab (summary data code: ukb-a-13) and Philip et al. (only UK Biobank summary statistics)^28^.

For the MR analysis, the exposure SNPs underwent several QC processes. We removed SNPs of the sex chromosomes, and those in the major histocompatibility complex region (chromosome 6:25–34 M) because of their strong pleotropic effects (Fig. S1)^39^. The major confounders for asthma and insomnia were reported to include body mass index, alcohol use, and physical activity^40–43^. To remove the exposure SNPs showing the strong association with the confounders and outcomes, we used PhenoScanner^44^. We examined the association of the exposure SNPs with the major confounders and outcomes, and excluded the SNPs associated at the genome-wide significance (*P* < 5 × 10^–8^). We then replaced missing SNPs that were not in the summary statistics with proxy SNPs (r^2^ > 0.8), and excluded palindromic SNPs (i.e. SNPs with alleles G and C, or A and T; MAF > 0.30)^45^. The detailed MR analysis study design and information of SNPs are shown in Fig. S1 and Tables S2–S4.

To assess the causal relationship between insomnia and asthma, we used inverse-variance weighted (IVW) estimate for the primary analysis^46^, and the weighted median^20^, MR-Egger regression (MR-Egger)^47^, and Mendelian randomization pleiotropy residual sum and outlier (MR-PRESSO)^48^ for the sensitivity analyses. The IVW method is the most basic MR method based on Wald ratio (the ratio of SNP-outcome to SNP-exposure effect), and the IVW analysis is calculated by the meta-analysis for all genetic variants included in the MR analysis. The IVW method is the most efficient MR method when all genetic variants satisfy all three assumptions, as follows: (1) the genetic variants are associated with the exposure, (2) the genetic variants have no association with the outcome except through the exposure, and (3) the genetic variants are not associated with confounding factors. However, if there are genetic variants showing the violation in any MR assumption, then it can raise a severe bias in MR analysis. All three MR methods including the weighted median, the MR-Egger, and the MR-PRESSO could correct the bias. The weighted median is unaffected by outliners, making the weighted median estimate insensitive to invalid genetic variants. Therefore, the weighted median provides an unbiased estimate of the causal effect even when up to 50% of the information comes from invalid genetic variants^20,49^. In contrast, the MR-Egger method uses all genetic variants even the invalid variants by estimating appropriate causal effects in the presence of pleiotropic effects. The MR-Egger permits the pleiotropy effect of genetic variant by allowing non-zero intercepts^50^. An intercept term that differs from zero indicates overall directional pleiotropy^51^. The fourth method, MR-PRESSO has an advantage over MR-Egger, in that it identifies directional pleiotropic outliers in MR testing and corrects for the bias derived from the directional pleiotropy by removing the outliers^48^. The heterogeneity of the MR analysis was evaluated using Cochran’s Q statistic in IVW and MR-Egger, and the pleiotropy was estimated using MR-Egger intercept and a global MR-PRESSO test. The entire MR analysis was performed by ‘TwoSampleMR’ package of R^50^.

### Cross-trait meta-analysis

For the cross-trait meta-analysis, we used R package Cross Phenotype Association (CPASSOC) which combined effect estimates and standard errors of GWAS summary statistics to test the hypothesis of association for a SNP between two traits^52^. We used a heterogenous version of CPASSOC (SHet). SHet is based on a fixed effect model and more powerful when there is a heterogenous effect present between studies, which is common in meta-analysis of different phenotype^21^. SHet uses the sample size for a trait as a weight instead of variance and accounts correlation due to overlapping or related subjects within and among different studies. In the cross-trait meta-analysis, we used the UK Biobank asthma and insomnia GWAS summary data from Neale Lab, and identified statistically significant SNPs (*P*_single trait_ < 0.05 and *P*_meta_ < 5 × 10^–8^). FUMA was used for identifying lead SNPs and performed the eQTL gene-mapping of SNPs^53^. Lead SNPs were identified as a subset of the significant SNPs that were in LD with each other at *r*^*2*^ < 0.1 within a 500 kb window.

### Calculation of genetic risk score (GRS) for insomnia and asthma

For the calculation of the GRS for insomnia (GRS_insomnia_), 202 insomnia-associated SNPs at genome-wide significance level (*P* < 5 × 10^–8^)^28^ were selected. A total of 163 SNPs were used for the GRS calculation after same QC processes as MR analysis (Table S3). For the calculation of GRS for asthma (GRS_asthma_), asthma-associated SNPs at genome-wide significance (*P* < 5 × 10^–8^) were selected^30–38^. After linkage disequilibrium-based clumping (r^2^ < 0.1, > 250 kb), 168 SNPs were retained for GRS_asthma_ calculation (Table S1).

The weighted score was calculated using the following equation^54^:

$$ Weighted\,score = \, \upbeta_{{1}} \times SNP_{{1}} + \, \upbeta_{{2}} \times SNP_{{2}} + \cdots \upbeta_{{\text{n}}} \times SNP_{{\text{n}}} , $$

where, β is the coefficient that represents the association between each SNP and phenotype. To calculate the weighted GRS for insomnia, we used the β coefficients of insomnia-associated SNPs from the GWAS summary statistics of the UK Biobank and 23andMe^28^ (Table S3). To calculate the weighted GRS for asthma, we used the β coefficients of asthma SNPs from 9 asthma GWASs, and selected the most significant β coefficient value if a SNP has multiple β coefficients from GWASs (Table S1).

The weighted score was rescaled to reflect the number of phenotype-increasing alleles as^54^:

$$ Weighted \,GRS= \frac{Weighted \,score\, \times \,number \,of\, available\, SNPs}{{Sum\, of\, the \,\beta\, coefficients\,of\,available \,SNPs}}. $$

### Interaction analysis of insomnia as a risk factor of asthma

For the investigation of the gene-environment interaction (GRS_asthma_ × *E*) of insomnia on asthma, we performed a logistic regression analysis. Models were adjusted for covariates: age, age^2^, sex, PCs, and batch size (array). To control for possible confounding effects^24^, interaction terms for GRS with age, age^2^, and sex as well as interaction terms for insomnia (*E*) with age, age^2^, and sex were also included. The following equation was used:

$$ {\text{Asthma}}=\upbeta_{0} + \, \upbeta_{{1}}\, GRS + \, \upbeta_{{2}} \,E + \, \upbeta_{{3}}\, GRS \times E + \,\upbeta_{{4}}\, Age + \, \upbeta_{{5}} \,Age^{{2}} + \, \upbeta_{{6}} \,Sex + \, \upbeta_{{7}} \,GRS \times Age + \, \upbeta_{{8}}\, GRS \times Age^{{2}} + \upbeta_{{9}} \,GRS \times Sex + \, \upbeta_{{{1}0}}\, E \times Age + \, \upbeta_{{{11}}}\, E \times Age^{{2}} + \upbeta_{{{12}}}\, E \times Sex + \upbeta_{{{13}}}\, Array + \sum\nolimits_{i = 1} {\upbeta_{{PC_{i} }} PC_{i} + \varepsilon .} $$

### Statistical analysis

SNP quality control and weighted GRS calculation performed using PLINK v.1.9.0^27^. Student’s *t*-tests, analysis of variance (ANOVA), and regression analyses were performed using IBM SPSS Statistics for Windows, version 25.0. We performed Mendelian randomization, cross-trait meta-analysis, variable standardisation, quantile–quantile (Q–Q) plots, heatmaps, and histograms in R stats package version 3.5.3 (www.r-project.org). The ‘TwoSampleMR’ package was used for MR analysis, ‘CPASSOC’ was used for cross-trait meta-analysis, ‘qqman’ package was used for plotting QQ plots, and ‘ggplot2’ package was used for plotting heatmaps and histograms.

## Results

After the QC and sample selection, the total study population was 272,154, including 38,641 cases of asthma and 233,513 controls (Table 1). In the asthma case group, 57.8% were women, 47.8% had allergic diseases, and 45.3% were on asthma-related drugs. The wheeze percentage, blood eosinophil count, neuroticism score and ratio of major depressive disorder (MDD) were higher in the asthma case group than in the control group (*P* < 0.01).

**Table 1:** Basic characteristics of participants with and without asthma (case and control groups).

	Case (n = 38,641)	Control (n = 233,513)
Age, years	56 (8.2)	57 (7.9)
**Sex**		
Women	22,335 (57.8%)	142,307 (52.6%)
Men	16,306 (42.2%)	128,010 (47.4%)
Asthma medication use	17,489 (45.3%)	NA
Hayfever, eczema or allergic rhinitis	18,461 (47.8%)	NA
Wheeze^a,b,c^	24,576 (64.6%)	27,775 (12.1%)
Blood eosinophil count^a,b^	0.218 (0.176)	0.156 (0.125)
Neuroticism score^a,b^	4.52 (3.36)	3.94 (3.21)
MDD^a,b^	3216 (12.81%)	11,368 (6.79%)
**Sleeplessness/insomnia**^**b**^		
Never/rarely	8215 (21.2%)	58,474 (25.1%)
Sometimes	18,032 (46.7%)	111,907 (47.9%)
Usually	12,394 (32.1%)	63,132 (27.0%)

All data are presented as mean ± standard deviation or numbers (%).

*NA* not applicable, *MDD* major depressive disorder.

^a^Participants available with this data were analysed.

^b^Significant difference between case and control (*P* < 0.01).

^c^Wheezing or whistling in the chest in the last year.

More than 78% of the participants suffered from insomnia, as they answered ‘sometimes’ or ‘usually’ to the question ‘Do you have trouble falling asleep at night or do you wake up in the middle of the night?’ (Table 1). The proportion of participants who answered ‘never/rarely’ was lower in the asthma case group (21.2%) than in the control group (25.1%), and the proportion of ‘usually’ was higher in the asthma case group (32.1%) than in the control group (27.0%; *P* = 5.44 × 10^–111^; Table 1). Similarly, the proportion of asthma cases was lower in the groups of participants who answered ‘never/rarely’ (12.3%) than those who answered ‘sometimes’ (13.9%) and ‘usually’ (16.4%; *P* = 1.14 × 10^–109^; Table S5). The neuroticism score and ratio of MDD related to insomnia^55,56^ increased as the degree of insomnia increased, and sleep duration decreased as the degree of insomnia increased (*P* < 0.01 for all three variables). Based on these results, we considered participants who answered ‘never/rarely’, ‘sometimes’, and ‘usually’ as normal, those with mild insomnia, and those with moderate-to-severe insomnia, respectively.

### Genetic evidence for causal association between asthma and insomnia

To confirm the relation between insomnia and risk of asthma, we performed a logistic regression analysis using insomnia questionnaires. The analysis confirmed that insomnia had a positive association with asthma (odds ratio [OR] 1.20 for one-unit increase of insomnia category, 95% confidence interval [CI] 1.18–1.21; *P* = 4.32 × 10^–116^). Pair-wise analysis revealed that participants who answered ‘sometimes’ showed 1.16 times higher OR (95% CI 1.12–1.19; *P* = 1.08 × 10^–23^) than those who answered ‘never/rarely’. Further, the participants who answered ‘usually’ showed 1.42 times higher OR (95% CI 1.38–1.46; *P* = 1.06 × 10^–111^) than those who answered ‘never/rarely’ (Fig. S2).

Observational associations between insomnia and the risk of asthma are often prone to bias from confounding and reverse causality. Therefore, we performed an MR analysis using insomnia-associated SNPs to confirm the causal effects of insomnia on asthma. First, we used TAGC asthma GWAS summary data that was completely independent of the sample used in insomnia GWAS meta-analysis (UK Biobank and 23andMe). Three methods, IVW, weighted median, and MR-PRESSO, proved that insomnia had a causal effect on the risk of asthma (IVW: OR = 1.10 for one-unit increase in log odds of liability to insomnia, 95% CI 1.03–1.18, *P* = 4.97 × 10^–3^; weighted median: OR 1.10 for one-unit increase in log odds of liability to insomnia, 95% CI 1.00–1.20, *P* = 0.047; MR-PRESSO: OR 1.10 for one-unit increase in log odds of liability to insomnia, 95% CI 1.03–1.18, *P* = 5.86 × 10^–3^; Table 2). The OR was expressed the average change for the outcome (asthma) per 2.72-fold increase in the prevalence of the exposure (insomnia)^57^. Additionally, the analyses did not show heterogeneity (*P* > 0.20) and directional pleiotropy (MR-Egger intercept *P* = 0.72; MR-PRESSO global test *P* = 0.23). To validate the causal effect of insomnia on the risk of asthma, we used UK Biobank asthma GWAS summary data from Neale Lab. Here as well, IVW and weighted median showed that insomnia was a risk factor for asthma (IVW: OR 1.01 for one-unit increase in log odds of liability to insomnia, 95% CI 1.01–1.02, *P* = 3.32 × 10^–9^; weighted median: OR 1.01 for one-unit increase in log odds of liability to insomnia, 95% CI 1.01–1.02, *P* = 1.41 × 10^–6^; Table S6). However, heterogeneity and directional pleiotropy were significant by MR estimates (*P* < 3.53 × 10^–6^ for heterogeneity; MR-PRESSO global test *P* < 0.001 for directional pleiotropy). MR-PRESSO identified two outlier SNPs and removed outlier SNPs for corrected causal estimate. Also in this result, insomnia was risk factor for asthma (MR-PRESSO: OR = 1.01 for one-unit increase in log odds of liability to insomnia, 95% CI 1.01–1.02, *P* = 5.22 × 10^–10^; Table S6) and no sign of a significant distortion in the causal estimate before and after MR-PRESSO correction (MR-PRESSO distortion test *P* = 0.853).

**Table 2:** Bidirectional Mendelian randomization analyses of insomnia on asthma.

Exposure	Outcome	Method	n SNPs	OR	95% CI	*P-*value
Insomnia	Asthma^a^	IVW	113	1.10	1.03–1.18	4.97 × 10^–3^
		Weighted median	113	1.10	1.00–1.20	0.047
		MR-Egger	113	1.03	0.72–1.48	0.87
		MR-PRESSO	113	1.10	1.03–1.18	5.86 × 10^–3^
Test for heterogeneity: *P* = 0.22 in MR-Egger and *P* = 0.20 in IVW						
Test for direction pleiotropy: MR-Egger intercept = 2.73 × 10^–3^, SE = 7.60 × 10^–3^, *P* = 0.72 and MR-PRESSO global test *P* = 0.23						
Asthma	Insomnia^b^	IVW	152	1.00	0.99–1.01	0.79
		Weighted median	152	1.00	0.99–1.01	0.85
		MR-Egger	152	1.00	1.00–1.00	0.88
		MR-PRESSO	150 ^c^	1.00	1.00–1.00	0.95
Test for heterogeneity: *P* = 1.93 × 10^–5^ in MR-Egger and *P* = 2.42 × 10^–5^ in IVW						
Test for directional pleiotropy: MR-Egger intercept = − 4.81 × 10^–5^, SE = 3.99 × 10^–4^, *P* = 0.90 and MR-PRESSO global test *P* < 0.001						

^a^Summary data code: ebi-a-GCST006862.

^b^Summary data code: ukb-a-13.

^c^MR-PRESSO horizontal pleiotropy corrected causal estimate.

Next, we tested the reverse causality, by considering asthma as an exposure and insomnia as an outcome in the MR analysis. The asthma-associated SNPs were tested for the causal effect of asthma on the risk of insomnia using UK Biobank insomnia GWAS summary data (the Neale Lab summary data). The MR analysis for the bidirectional test could not prove that asthma had a causal effect on insomnia (Table 2), suggesting that the relationship between insomnia and asthma was unidirectional instead. Since two GWASs of the UK Biobank and 23andMe^28^ and the Neal lab summary statistics used the different criteria of insomnia, we re-analyzed the bidirectional test of asthma on insomnia using a summary statistics provided by Philip et al.^28^, which used the same criteria for insomnia as the UK Biobank and 23andMe. As shown in Table S6, the MR results were almost similar to those obtained by using the Neal lab data.

To further validate the results of the bidirectional MR analysis, we calculated GRS_insomnia_ and GRS_asthma_ and tested their associations with asthma and insomnia separately by logistic regression. First, the GRS_insomnia_ variables followed a normal distribution (Fig. S3) and ranged from minimum 123.62 to maximum 193.13 with mean ± SD of 157.65 ± 7.48. In the logistic regression, the GRS_insomnia_ was significantly associated with insomnia as expected (β [SE] = 0.0060 [0.0002], *P* = 2.13 × 10^–239^; Table S7). Also, there was a positive significance association observed between GRS_insomnia_ and asthma (OR = 1.006, 95% CI 1.004–1.007, *P* = 7.52 × 10^–15^), indicating that one-unit increase in GRS_insomnia_ corresponded to a 0.6% increase in the risk of asthma. This result may be biased because the GRS_insomnia_ of the UK Biobank sample was calculated using weights from insomnia GWAS meta-analysis of UK Biobank and 23andMe. Therefore, we calculated unweighted GRS_insomnia_ to avoid internal weight, and performed the logistic regression. As same as weighted GRS_insomnia_, there was a positive significance association observed between unweighted GRS_insomnia_ and asthma (OR 1.005, 95% CI 1.004–1.007, *P* = 6 × 10^–14^). Second, the GRS_asthma_ also followed a normal distribution (Fig. S4) and ranged from 101.89 to 170.65 with mean ± SD of 133.58 ± 7.35. In the logistic regression, the GRS_asthma_ was significantly associated with asthma as expected (OR 1.061 for one-unit increase of GRS_asthma_, 95% CI 1.059—1.063, *P* < 1 × 10^–300^; Table S8). However, GRS_asthma_ was not associated with insomnia (*P* = 0.37; Table S8), thereby confirming the results of the bidirectional MR analysis.

### Cross-trait meta-analysis between asthma and insomnia

The genetic correlation between asthma and insomnia was previously reported in the range of 0.17 to 0.26^28,58^, therefore we performed the cross-trait meta-analysis to identify the shared genes between asthma and insomnia. We used a heterogenous version of CPASSOC (SHet) for the cross-trait meta-analysis, using the UK Biobank asthma and insomnia GWAS summary data from Neale Lab. As a result, we identified 28 lead SNPs having a shared effect (*P*_single trait_ < 0.05 and *P*_meta_ < 5 × 10^–8^), and 41 cis-eQTL genes were mapped from the 28 lead SNPs in GTEx v8^59^ through FUMA (Tables S9, S10). Among the 41 genes, 18 were reported in asthma GWASs, 6 were reported in insomnia GWASs, and 3 were reported in both GWASs, based on GWAS Catalog.

### Gene-environment interactions of GRS_asthma_ and insomnia on asthma

To investigate whether insomnia affects asthma through interaction with genetic factors, we performed a gene-environment interaction study using GRS_asthma_. In logistic regression analysis, GRS_asthma_ showed a significant positive association with asthma, and an increase of one unit in GRS_asthma_ corresponded to a 6.1% increase in the risk of asthma (Table S11).

**Figure 1:**
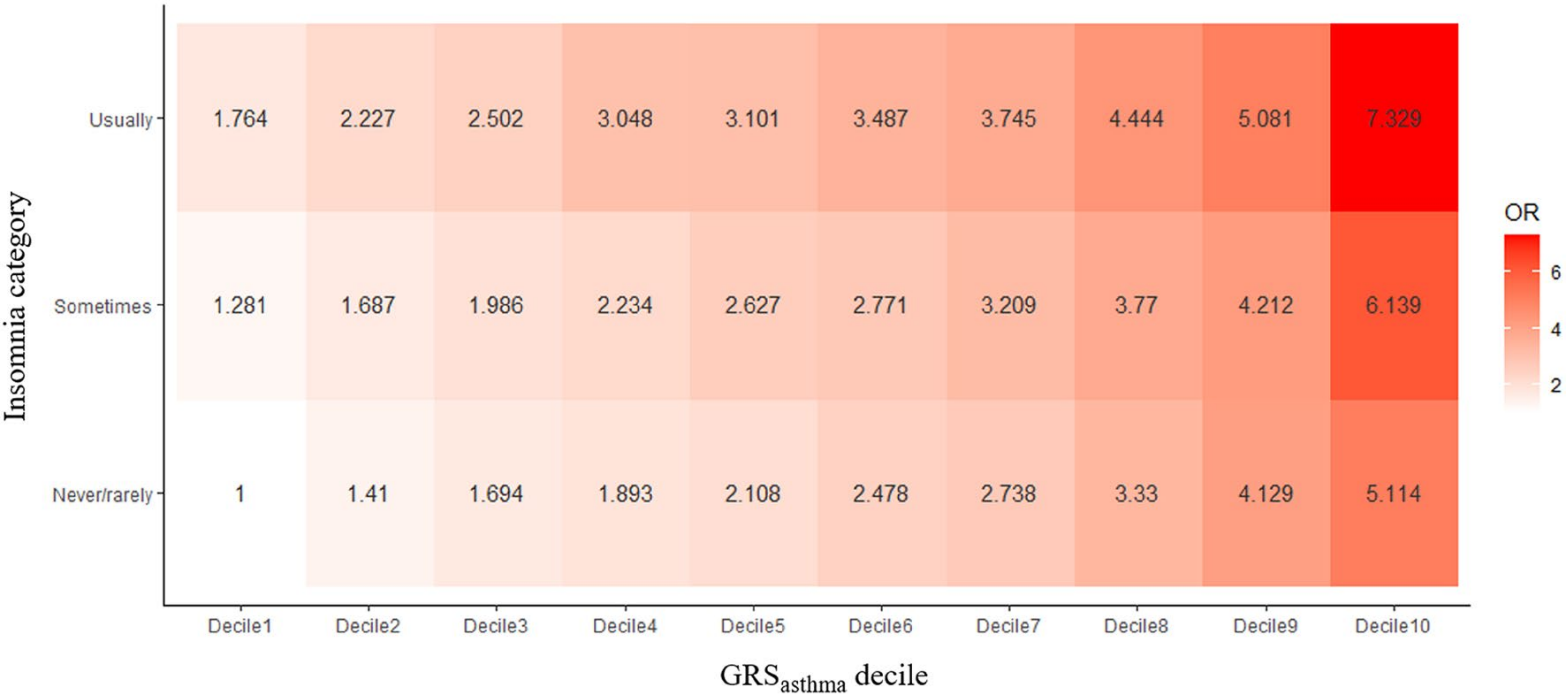
Odds ratio (OR) heatmap of asthma between GRS_asthma_ and insomnia categories. The OR of asthma was compared on the basis of the OR of the subset with the lowest GRS_asthma_ and ‘never/rarely’ category of insomnia, and adjusted with respect to age and sex. All subsets and reference subsets show significant differences in the OR (P < 0.05).

To examine the effect of GRS_asthma_ and insomnia on asthma, a total of 30 subsets were formed by categorising participants on the basis of GRS_asthma_ deciles and insomnia categories. Based on the subset with the lowest GRS_asthma_ and ‘never/rarely’ response, we calculated the OR of each subset for the risk of asthma using logistic regression (Fig. 1). In all subsets, higher GRS_asthma_ was associated with a higher risk of asthma in the same insomnia category. Additionally, more severe insomnia (‘usually’) was associated with a higher risk of asthma in the same GRS_asthma_ decile. The subset with the highest GRS_asthma_ and severe insomnia (‘usually’) showed an approximately seven times higher (OR 7.33, 95% CI 6.52–8.24, *P* = 2.17 × 10^–242^) risk of asthma than the subset with lowest GRS_asthma_ and normal response (‘never/rarely’). These results indicate that GRS_asthma_ is a good indicator of genetic risk of asthma, and that insomnia acts as a risk factor for asthma at any genetic risk score.

Next, interaction analysis was performed to determine whether GRS_asthma_ and insomnia affected asthma through interaction using a logistic regression model by adjusting for age, age^2^, sex, batch size, and 10 PCs as covariates. The interaction between GRS_asthma_ and insomnia was significant for asthma (OR 0.995, 95% CI 0.993–0.997, *P* = 7.22 × 10^–6^; Table 3). Further, the interaction between GRS_asthma_ and insomnia was tested by adjusting the interaction terms of age, age^2^, and sex to exclude confounding effects; the results were still significant (OR 0.997, 95% CI 0.995–0.999, *P* = 1.68 × 10^–3^; Table 3). Since both variables of GRS_asthma_ and insomnia were risk factors to the asthma incidence, we had expected that the interaction might be also a risk factor. Surprisingly, the effect of interaction i.e. GRS_asthma_ × insomnia reduced the risk of asthma unexpectedly (OR < 1). To explore this, two more tests were performed.

**Table 3:** Intercation effect of GRS_asthma_ with insomnia on asthma.

Insomnia × GRS_asthma_^a^		Insomnia × GRS_asthma_^a,b^	
*P-*value	OR (95% CI)	*P-*value	OR (95% CI)
7.22 × 10^–6^	0.995 (0.993–0.997)	1.68 × 10^–3^	0.997 (0.995–0.999)

^a^Model was adjusted for covariates: age, age^2^, sex, batch size, and 10 principal components.

^b^Interaction terms were adjusted for covariates: age, age^2^, and sex.

**Figure 2:**
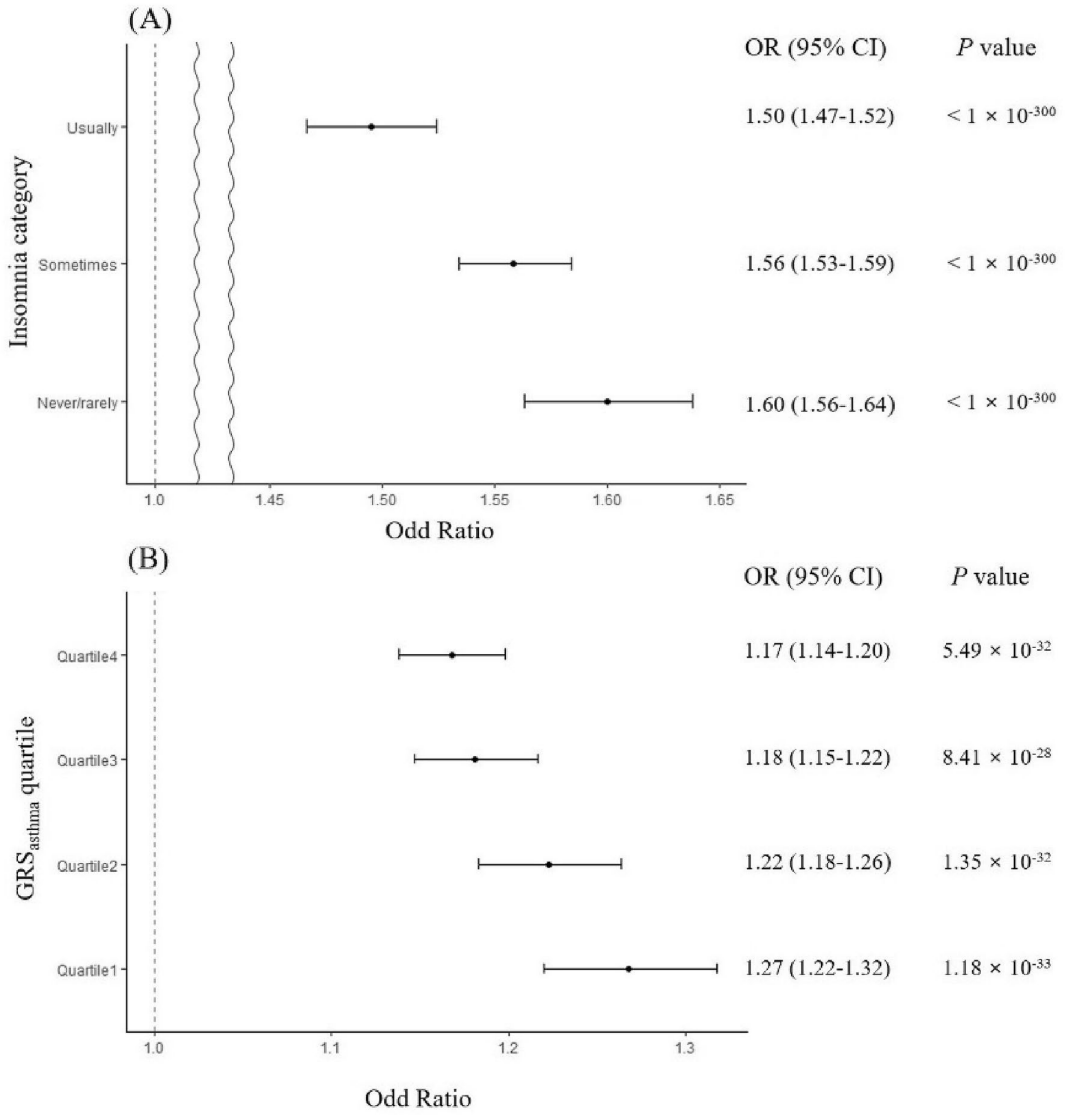
Effect of GRS_asthma_ and insomnia on asthma on the basis of insomnia categories and GRS_asthma_ quartiles. The odds ratio (OR) of asthma is plotted as dots ± 95% confidence interval. (**A**) The association between asthma and GRS_asthma_ by insomnia categories by considering age, age^2^, sex, batch size, and 10 principle components. The GRS_asthma_ is scaled to mean = 0 and standard deviation = 1 to facilitate interpretation. (**B**) The variation in OR for asthma on insomnia by GRS_asthma_ quartiles after considering age and sex.

First, all subjects were categorised by their insomnia categories of ‘never/rarely’, ‘sometimes’, and ‘usually’. Effect of GRS_asthma_ on asthma was calculated by logistic regression within these categories. As shown in Fig. 2A, the effect size of GRS_asthma_ increased as insomnia severity decreased. Second, all subjects were categorised into quartiles based on individual GRS_asthma_, and effect size of insomnia on asthma was calculated by logistic regression within the quartiles. Here, the effect size of insomnia increased as GRS_asthma_ decreased (Fig. 2B).

The UK Biobank data provides the Electronic Health Records (EHR) data of ICD-9/10 code (UK Biobank data-field 41,202–41,205, 41,270–41,271) for the classification of asthma patients (number of asthma cases: 24,366 from EHR data). To validate the results above, we conducted all analyses for the association of insomnia on asthma and also the interaction of insomnia with GRS_asthma_ on asthma (Tables S11, S12). While there were minor differences of *P-*value and OR between results from self-reported data and EHR data, both results were statistically significant and showed the same direction of effect for each analysis. Additionally, we analyzed the effect of GRS_asthma_ on asthma according to the insomnia category as well as the effect of insomnia on asthma according to the GRS_asthma_ quartile category, shown in Fig. 2. Similarly, there were minor differences of *P-*value and OR between results from self-reported data and EHR data, but both results were statistically significant and showed the same direction of effect for each analysis (Fig. S5).

## Discussion

In this study, we employed MR analysis and found that insomnia acted as a risk factor for asthma; however, the opposite was not true. Furthermore, the cross-trait meta-analysis identified 28 shared genetic loci between asthma and insomnia, and the analysis of GRS_asthma_ × insomnia interaction showed that insomnia interacted with asthma-associated SNPs to affect the risk of asthma.

Previous studies have been^28^ unable to establish a directional association between insomnia and asthma, and a possible reason may be the small sample size (2,237 individuals). In this study, we used GWAS summary statistics obtained from large sample sizes (Neale lab summary data code: ukb-a-446 with 336,782 individuals; Neale lab summary data code: ukb-a-13 with 336,965 individuals; and TAGC summary data code: ebi-a-GCST006862 with 127,669 individuals). The large sample sizes allowed us to prove the unidirectional causality between insomnia and asthma. This is in contrast with previous epidemiological studies that have indicated a bidirectional causality between the two diseases^10–12^. However, such observations are often the case with MR analyses; for instance, causality between moderate alcohol intake and cardiovascular disease, which is observed in conventional epidemiological analyses, could not be proved by MR analysis^60^.

There are several possible mechanisms by which insomnia may act as a risk factor for asthma. The most studied one is the effect of insomnia on the immune system. Insomnia has been associated with chronic inflammation and pro-inflammatory cytokine production change^13^. IL-6, well known biomarker for asthma, increased nocturnally secretion in insomnia patients^61^. In addition, the activation of NF-κB and level of high sensitivity C-reactive protein (CRP) increases during lack of sleep^62,63^. In fact, activation of NF-κB can affect asthma through inflammatory protein regulation^64,65^, and the increased level of high sensitivity CRP is also associated with airflow obstruction and airway inflammation^66^. Another possible mechanism is neuro-immune defects, which is mediated by the microbiome-based brain-gut axis, may contribute to asthma due to sleep disorder. Moreover, it has been widely investigated that gene and lifestyle may affect the neuro-immune functions through brain-gut axis^67–69^. Additionally, sleep disorder may trigger the aberrations of RNA modifications and further cause the dysregulations of gene expression. The recent progress in N4-Acetylcytidine on RNA expression is also playing a key role on the development of asthma^70^. Although we did not know which one of the above mechanisms belong to the primary one, those would be considered as the underlying mechanisms explaining the causal effect of insomnia on the asthma incidence. In addition, those genetic factors involved in these pathways would express the effect in the MR analysis as the vertical pleiotropy.

Through the cross-trait meta-analysis, we identified shared genes between asthma and insomnia. In addition, we investigated whether shared genes support the implication of immune pathway between asthma and insomnia. Among the shared genes, three genes, *ITPR3, HEXIM1*, and *IQCH,* were found to be reported in both GWASs of asthma and insomnia (Table S10), and two genes, *ITPR3* and *HEXIM1* appear to be involved in the inflammation process. *ITPR3* control the intracellular Ca^2+^ levels which determined the short and long-term function of T lymphocytes^71^, and *HEXIM1* regulate the innate immune response through the cGAS-STING pathway^72^. Additional shared genes such as *SMAD3, LIME1, IL13* and *IL1R2,* etc., were also associated with inflammation process. Compared with the above inflammatory mechanisms considered as vertical pleiotropy, these shared genes could work in the pathway of either horizontal or vertical pleiotropy. Further study warrants to determine whether these shared genes belong to vertical pleiotropy.

We also found that the GRS_asthma_ × insomnia interaction affected the risk of asthma. However, the interaction unexpectedly reduced the risk of asthma (OR < 1), although individually these risk factors greatly increased the risk of asthma. As insomnia worsened, the genetic effect of GRS_asthma_ became weaker, and as GRS_asthma_ increased, the environmental effect of insomnia became weaker. Possibly, the two factors interacted to alleviate the risk of asthma. Thus, we speculate that both risk factors share pathways through which the factors affected the risk of asthma. Further, the combined effect of two risk factors may be reduced if the interaction worked in a similar way as in complementation tests, which are defined as tests that indicate the disappearance of a mutation phenotype in a hybrid of two homozygous mutants with the same phenotype having a mutation in two different genes of the same pathway.

This study has several limitations. First, we used participants of only European ancestry, therefore the effect of insomnia on asthma may be ethnically different. Second, we used self-reported questionnaire data about asthma and insomnia from the UK Biobank, which may be prone to biases and inaccuracies. Therefore, we utilised asthma-related variables (e.g. wheeze percentage, blood eosinophil count) and insomnia-related variables (e.g. sleep duration, MDD percentage) to support the validity of the data used. Also, we performed the association and interaction analysis of insomnia on asthma again with the criteria of asthma case from EHR data i.e. ICD 9/10 code, and confirmed that the results were similar to those from the self-reported data. Third, in the UK, around 9 million individuals were invited to participate about UK Biobank, but only about 5% of them responded, so the UK Biobank sample is not representative of the UK population as a whole^73^. For example, UK Biobank participants were more likely to be older, to be female, and to live in less socioeconomically deprived areas, were less likely to be obese, to smoke, and to drink alcohol, and had fewer self-reported diseases, compared to the general population^74^. Therefore, the selection bias might arise^75^. However, selection biases are known to be problematic for MR estimation only if the effect of selection is large, and the size of selection will generally be unknown^76^. Also, the effect of selection bias has been suggested to be less than that of other bias (confounding effect or pleiotropic effect)^77^. In conclusion, our results highlight the importance of insomnia as a risk factor for asthma, and warrant further investigations to elucidate the underlying mechanisms that may explain the relationship between insomnia and asthma.

## Supplementary Information

Supplementary Information 1.

Supplementary Information 2.
