# Supplementary material for: Characterisation of insomnia as an environmental risk factor for asthma via Mendelian randomization and gene environment interaction
Source: Sci Rep. 2021 Nov 8;11:21813. doi: 10.1038/s41598-021-01291-6 (PMC8576024; doi:10.1038/s41598-021-01291-6)
Supplement: Supplementary file 2 — Supplementary Information 2. [file 41598_2021_1291_MOESM2_ESM.docx]

**Characterisation of insomnia as an environmental risk factor for asthma via Mendelian randomization and gene-environment interaction**

Dong Jun Kim^1†^, Tae-Woong Ha^2†^, Hae Un Jung^1^, Eun Ju Baek^1^, Won Jun Lee^2^, Han Kyul Kim^2^, Ji-One Kang^2^, Sungho Won^3^, Ji Eun Lim^2*^, and Bermseok Oh^1,2*^

^1^Department of Biomedical Science, Graduate School, Kyung Hee University, Seoul, Korea

^2^Department of Biochemistry and Molecular Biology, School of Medicine, Kyung Hee University, Seoul, Korea

^3^Department of Public Health Science, Seoul National University, Seoul, Korea

^†^These authors contributed equally to this work.

***Corresponding authors**:

Ji Eun Lim

Department of Biochemistry and Molecular Biology, School of Medicine, Kyung Hee University, Seoul, South Korea.

Phone: +82 2-961-0617

E-mail: [jelim@khu.ac.kr](mailto:jelim@khu.ac.kr)

Bermseok Oh

Department of Biochemistry and Molecular Biology, School of Medicine, Kyung Hee University, Seoul, South Korea.

Phone: +82 2-961-0617

E-mail: [ohbs@khu.ac.kr](mailto:ohbs@khu.ac.kr)

Supporting Information

Asthma case group: Asthma case group constituted participants who answered ‘asthma’ when asked, ‘Has a doctor ever told you that you have had any of the following conditions?’ (data field 6152). Individuals who answered ‘emphysema/chronic bronchitis’ for the same question (data field 6152) or those who had been diagnosed with COPD by a doctor (data fields 22130, 22150) were excluded.

Asthma control group: The control group consisted of individuals who had not been diagnosed with asthma, rhinitis, eczema, allergy, and emphysema/chronic bronchitis (data field 6152). Further, participants who did not have the data for the following were also in the control group: diagnosed with hay fever or allergic rhinitis by a doctor (data field 22126), age of diagnosis with hay fever or allergic rhinitis (data field 22146), diagnosed with emphysema (data field 22128), age of diagnosis with emphysema (data field 22148), diagnosed with chronic bronchitis (data field 22129), age of diagnosis with chronic bronchitis (data field 22149), diagnosed with COPD (data field 22130), age of diagnosis with COPD (data field 22150), and ICD-10 J40-47 (data fields 41202, 41204). Additionally, individuals who were medicated with asthma-related drugs were excluded.

Blood eosinophil count: The blood eosinophil count means the number of eosinophil in nanoliter, by calculating the proportion of eosinophil in white blood cells × white blood cell count per nanoliter (data field 30150). Eosinophils Number assay was performed on blood sample of each participant.

Major depressive disorder (MDD): We applied the criteria from past paper[^1^](#_ENREF_1) to classify MDD participants. The MDD case group consisted of individuals who had indicated that they were ‘depressed/down for a whole week (data field 4598) plus at least 2 weeks duration (data field 4609) plus ever seen a GP or psychiatrist for nerves, anxiety or depression (data field 2090 and 2010)’ or ‘ever anhedonia for a whole week (data field 4631) plus at least 2 weeks duration (data field 5375) plus ever seen a GP or psychiatrist for nerves, anxiety or depression (data field 2090 and 2010)’or ICD-10 F32-34, F38-39 (data fields 41202, 41204).

Neuroticism score: Neuroticism score was derived from 12 neurotic behavioral domains provided by the UK Biobank (data field 20127). Each participant had been asked to answer the questions below. The number of “Yes” answers to the questions was returned as a single integer score for each participant.

- data field 1920: “Does your mood often go up and down?”

- data field 1930: “Do you ever feel 'just miserable' for no reason?”

- data filed 1940: “Are you an irritable person?”

- data filed 1950: “Are your feelings easily hurt?”

- data filed 1960: “Do you often feel 'fed-up'?”

- data filed 1970: “Would you call yourself a nervous person?”

- data filed 1980: “Are you a worrier?”

- data filed 1990: “Would you call yourself tense or 'highly strung'?”

- data filed 2000: “Do you worry too long after an embarrassing experience?”

- data filed 2010: “Do you suffer from 'nerves'?”

- data filed 2020: “Do you often feel lonely?”

- data filed 2030: “Are you often troubled by feelings of guilt?”

|  | Never/rarely  (n=66,589) | Sometimes  (n=129,499) | Usually  (n=74,655) |
| --- | --- | --- | --- |
| Asthma | 8,215 (12.3%) | 18,032 (13.9%) | 12,394 (16.4%) |
| Sleep duration^a,b^ | 7.42 (0.96) | 7.31 (0.96) | 6.72 (1.24) |
| Neuroticism score^b^ | 2.80 (2.81) | 4.04 (3.15) | 5.14 (3.36) |
| MDD^b^ | 2,328 (4.43%) | 6,298 (6.86%) | 5,958 (12.37%) |
| Table S5. Basic characteristics of participants classified by insomnia states. All data are presented as mean ± standard deviation or numbers (%). All values in the table show significant differences between the categories (*P* < 0.01). MDD, major depressive disorder; a, sleep hours per day; b, participants available with this data were analysed. | | | |

| Exposure | Outcome | Method | n SNPs | OR | 95% CI | *P-*value |
| --- | --- | --- | --- | --- | --- | --- |
| Insomnia | Asthma^a^ | IVW  Weighted median  MR-Egger  MR-PRESSO | 163  163  163  161^b^ | 1.01  1.01  1.01  1.01 | 1.01-1.02  1.01-1.02  0.99-1.03  1.01-1.02 | 3.32 × 10^-9^  1.41 × 10^-6^  0.39  5.22 × 10^-10^ |
| Test for heterogeneity: *P* = 2.89 × 10^-6^ in MR-Egger and *P* = 3.53 × 10^-6^ in IVW | | | | | | |
| Test for directional pleiotropy:  MR-Egger intercept = 1.55 × 10^-4^, SE = 4.17 × 10^-4^, *P* = 0.71 and MR-PRESSO global test *P* < 0.001 | | | | | | |
| Asthma | Insomnia^c^ | IVW  Weighted median  MR-Egger  MR-PRESSO | 149  149  149  147^b^ | 1.00  1.00  1.00  1.00 | 0.99-1.01  0.98-1.02  0.98-1.03  0.99-1.01 | 0.95  1.00  0.94  0.92 |
| Test for heterogeneity: *P* = 1.80 × 10^-3^ in MR-Egger and *P* = 2.14 × 10^-3^ in IVW | | | | | | |
| Test for directional pleiotropy:  MR-Egger intercept = -1.40 × 10^-4^, SE = 1.16 × 10^-3^, *P* = 0.90 and MR-PRESSO global test *P* < 0.001 | | | | | | |
| Table S6. Bidirectional Mendelian randomization analyses of insomnia on asthma. a, summary data code: ukb-a-446; b, MR-PRESSO performed for corrected causal estimate after the exclusion of two outlier SNPs; c, summary data provided by Philip R., et al summary data | | | | | | |

| GRS_insomnia_ | | | | | |
| --- | --- | --- | --- | --- | --- |
| Insomnia | |  | Asthma | |  |
| *P*-value | Beta (SE) |  | *P*-value | OR (95% CI) |  |
| 2.13 × 10^-239^ | 0.0060 (0.0002) |  | 7.52 × 10^-15^ | 1.006 (1.004 - 1.007) |  |
| Table S7. Association analysis of GRS_insomnia_ with insomnia and asthma by logistic regression. Both models were adjusted for covariates: age, age^2^, sex, batch size and 10 PCs; SE, standard error; CI, confidence interval | | | | | |

| GRS_asthma_ | | | | | |
| --- | --- | --- | --- | --- | --- |
| Asthma | |  | Insomnia | |  |
| *P*-value | OR (95% CI) |  | *P*-value | Beta (SE) |  |
| < 1 × 10^-300^ | 1.061 (1.059 - 1.063) |  | 0.37 | 1.67 × 10^-4^ (1.85× 10^-4^) |  |
| Table S8. Association analysis of GRS_asthma_ with asthma and insomnia by logistic regression. Logistic regression models were adjusted for covariates: age, age^2^, sex, batch size and 10 PCs;  SE, standard error; CI, confidence interval | | | | | |

| GRS_asthma_ | |
| --- | --- |
| *P*-value | OR (95% CI) |
| < 1 × 10^-300^ | 1.055 (1.053-1.057) |
| Table S11. Association analysis of GRS_asthma_ with asthma classified by EHR data. Logistic regression model was adjusted for covariates: age, age^2^, sex, batch size and 10 PCs;  CI, confidence interval | |

| Insomnia × GRS_asthma_ ^a^ | |  | Insomnia × GRS_asthma_ ^a,b^ | |
| --- | --- | --- | --- | --- |
| *P-*value | OR (95% CI) |  | *P-*value | OR (95% CI) |
| 6.74 × 10^-5^ | 0.995(0.992-0.997) |  | 6.22 × 10^-3^ | 0.996(0.994-0.999) |
| Table S12. Intercation effect of GRS_asthma_ with insomnia on asthma classified by EHR data. a, model was adjusted for covariates: age, age^2^, sex, batch size, and 10 principal components; b, interaction terms were adjusted for covariates: age, age^2^, and sex. | | | | |


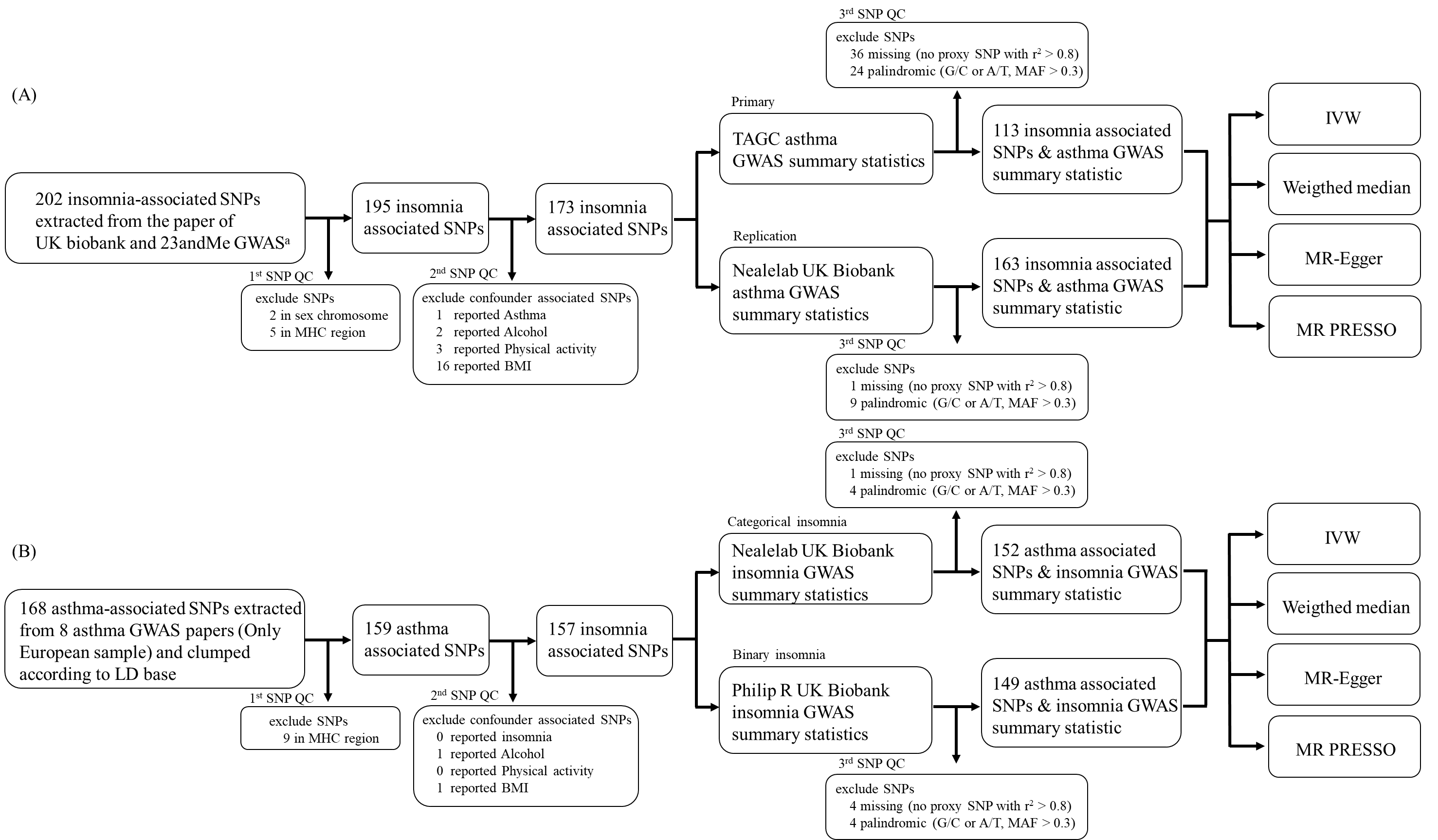


**Figure S1** Study design for the Mendelian randomisation (MR) analysis. A, MR analysis to test for insomnia as a risk factor for asthma; B, MR analysis to test for asthma as a risk factor for insomnia.


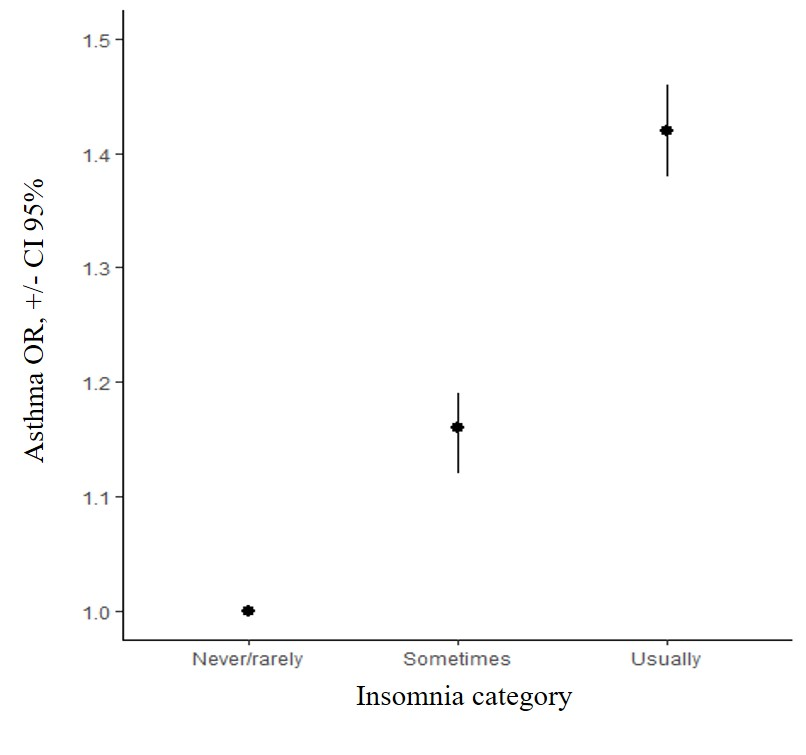


**Figure S2** Odds ratio (OR) for the risk of asthma in each insomnia category. The OR of each category, for determining the incidence of asthma, was calculated by comparing with the OR of ‘never/rarely’ category after adjustment for age and sex. CI, confidence interval.


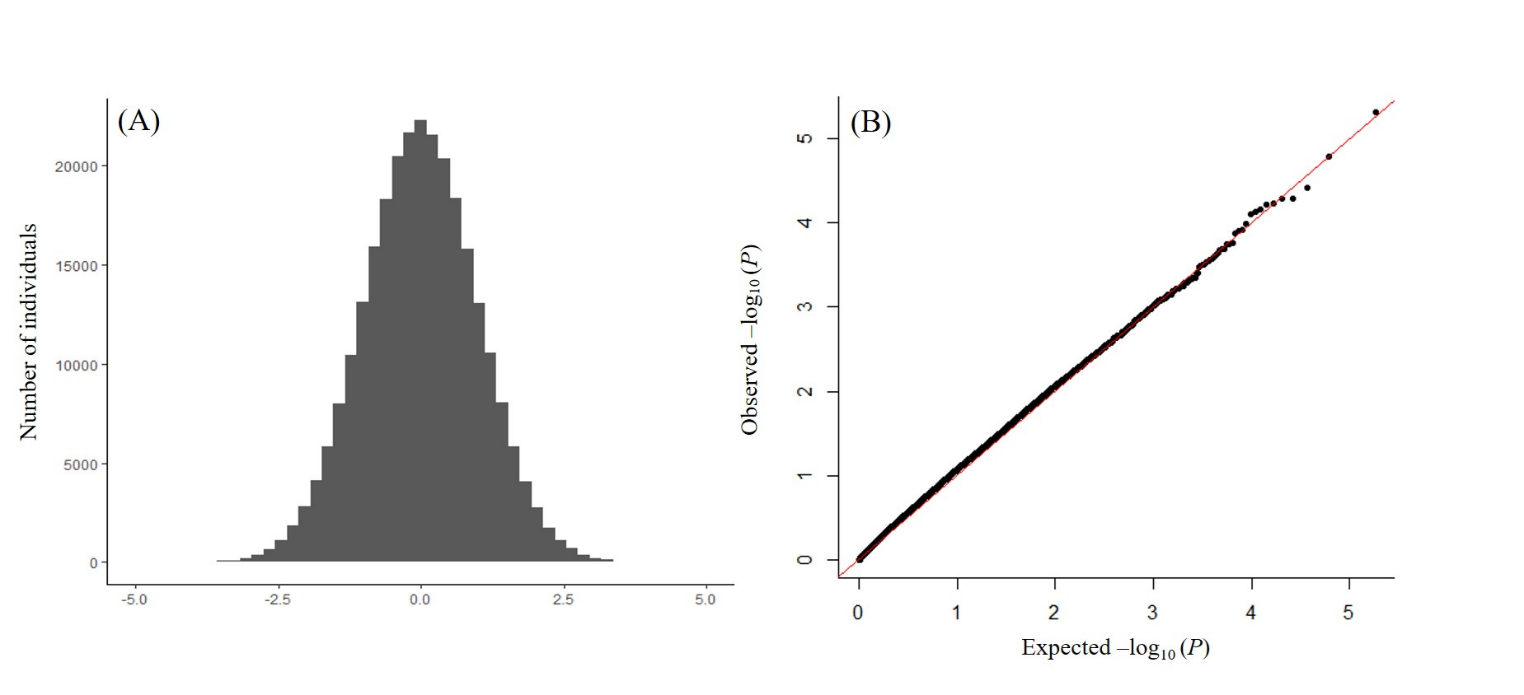


**Figure S3** Distribution of GRS_insomnia_ in participants from the UK Biobank. Figure (A) shows the distribution of the number of people according to GRS_insomnia_. The GRS_insomnia_ scaled to a mean of 0 and standard deviation 1 to facilitate interpretation. Figure (B) is Quantile-quantile (QQ) plot for GRS_insomnia_.


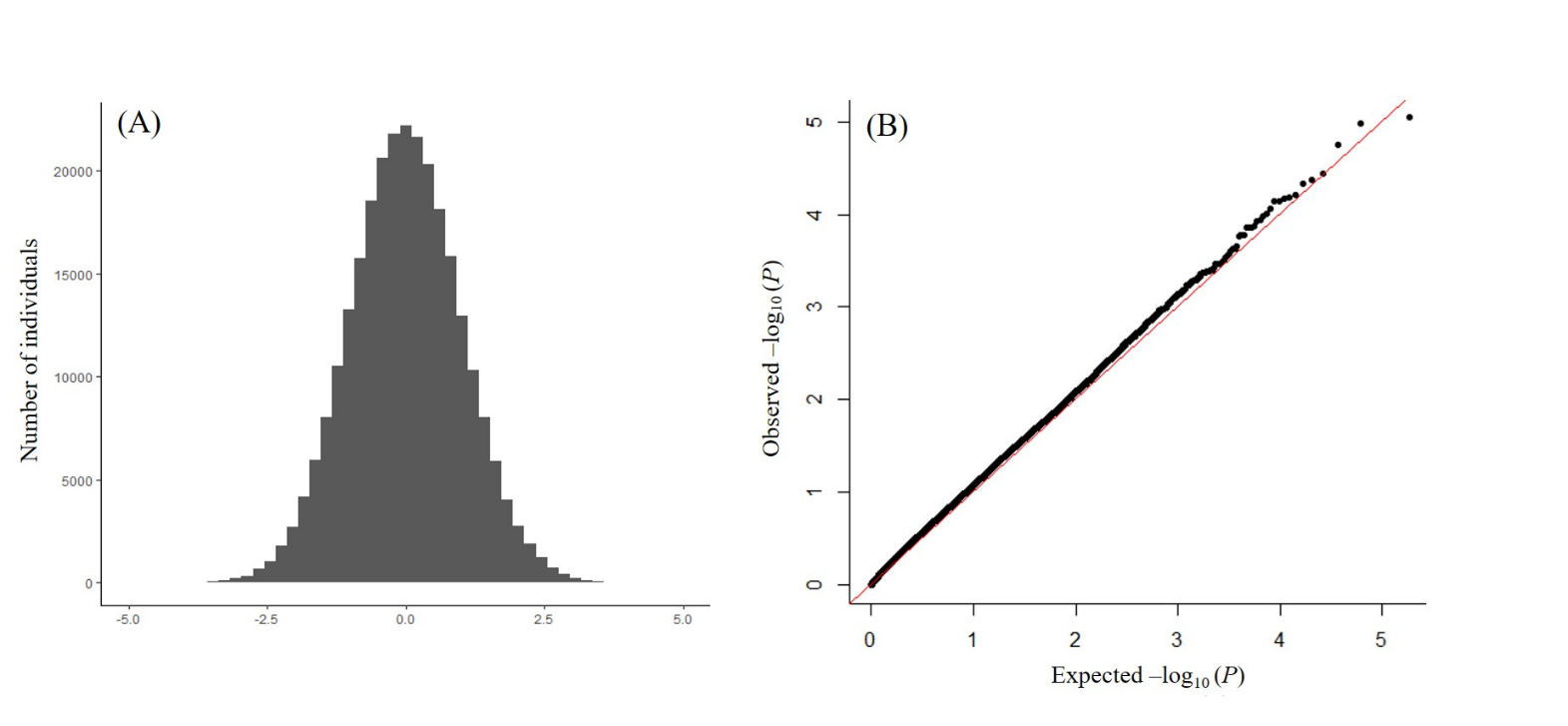


**Figure S4** Distribution of GRS_asthma_ in participants from the UK Biobank. Figure (A) shows the distribution of the number of people according to GRS_asthma_. The GRS_asthma_ scaled to a mean of 0 and standard deviation 1 to facilitate interpretation. Figure (B) is Quantile-quantile (QQ) plot for GRS_asthma_.


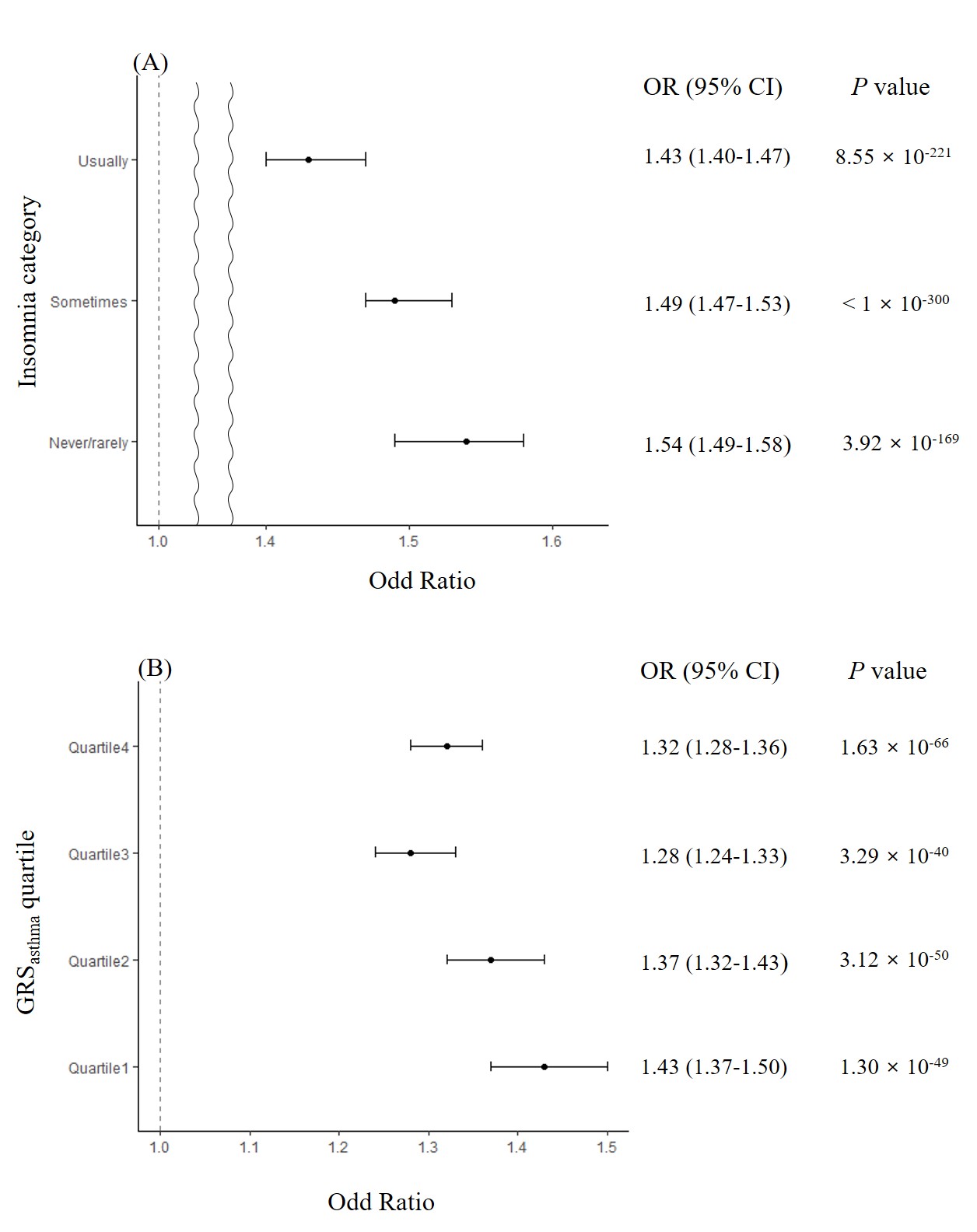


**Figure S5** Effect of GRS_asthma_ and insomnia on asthma on the basis of insomnia categories and GRS_asthma_ quartiles. The odds ratio (OR) of asthma is plotted as dots ± 95 % confidence interval. A, The association between asthma and GRS_asthma_ by insomnia categories by considering age, age^2^, sex, batch size, and 10 principle components. The GRS_asthma_ is scaled to mean = 0 and standard deviation = 1 to facilitate interpretation. B, The variation in OR for asthma on insomnia by GRS_asthma_ quartiles after considering age and sex.
